# A novel homozygous KCNQ3 loss‐of‐function variant causes non‐syndromic intellectual disability and neonatal‐onset pharmacodependent epilepsy

**DOI:** 10.1002/epi4.12353

**Published:** 2019-08-11

**Authors:** Anna Lauritano, Sebastien Moutton, Elena Longobardi, Frédéric Tran Mau‐Them, Giusy Laudati, Piera Nappi, Maria Virginia Soldovieri, Paolo Ambrosino, Mauro Cataldi, Thibaud Jouan, Daphné Lehalle, Hélène Maurey, Christophe Philippe, Francesco Miceli, Antonio Vitobello, Maurizio Taglialatela

**Affiliations:** ^1^ Division of Pharmacology, Department of Neuroscience University of Naples “Federico II” Naples Italy; ^2^ Reference Center for Developmental Anomalies, Department of Medical Genetics Dijon University Hospital Dijon France; ^3^ INSERM U1231, LNC UMR1231 GAD Burgundy University Dijon France; ^4^ Laboratoire de Génétique, Innovation en Diagnostic Génomique des Maladies Rares UF6254, Plateau Technique de Biologie CHU Dijon Dijon France; ^5^ Department of Medicine and Health Science “V. Tiberio” University of Molise Campobasso Italy; ^6^ Division of Pharmacology, Department of Science and Technology University of Sannio Benevento Italy; ^7^ Service de Neurologie Pédiatrique APHP, Hôpital Universitaire Bicêtre Le Kremlin‐Bicêtre France

**Keywords:** early‐onset epileptic encephalopathy, homozygous loss‐of‐function variant, intellectual disability, KCNQ3, next‐generation sequencing, nonsense‐mediated mRNA decay

## Abstract

**Objective:**

Heterozygous variants in *KCNQ2* or, more rarely, *KCNQ3* genes are responsible for early‐onset developmental/epileptic disorders characterized by heterogeneous clinical presentation and course, genetic transmission, and prognosis. While familial forms mostly include benign epilepsies with seizures starting in the neonatal or early‐infantile period, de novo variants in *KCNQ2* or *KCNQ3* have been described in sporadic cases of early‐onset encephalopathy (EOEE) with pharmacoresistant seizures, various age‐related pathological EEG patterns, and moderate/severe developmental impairment. All pathogenic variants in *KCNQ2* or *KCNQ3* occur in heterozygosity. The aim of this work was to report the clinical, molecular, and functional properties of a new *KCNQ3* variant found in homozygous configuration in a 9‐year‐old girl with pharmacodependent neonatal‐onset epilepsy and non‐syndromic intellectual disability.

**Methods:**

Exome sequencing was used for genetic investigation. KCNQ3 transcript and subunit expression in fibroblasts was analyzed with quantitative real‐time PCR and Western blotting or immunofluorescence, respectively. Whole‐cell patch‐clamp electrophysiology was used for functional characterization of mutant subunits.

**Results:**

A novel single‐base duplication in exon 12 of *KCNQ3* (NM_004519.3:c.1599dup) was found in homozygous configuration in the proband born to consanguineous healthy parents; this frameshift variant introduced a premature termination codon (PTC), thus deleting a large part of the C‐terminal region. Mutant KCNQ3 transcript and protein abundance was markedly reduced in primary fibroblasts from the proband, consistent with nonsense‐mediated mRNA decay. The variant fully abolished the ability of KCNQ3 subunits to assemble into functional homomeric or heteromeric channels with KCNQ2 subunits.

**Significance:**

The present results indicate that a homozygous *KCNQ3* loss‐of‐function variant is responsible for a severe phenotype characterized by neonatal‐onset pharmacodependent seizures, with developmental delay and intellectual disability. They also reveal difference in genetic and pathogenetic mechanisms between *KCNQ2*‐ and *KCNQ3*‐related epilepsies, a crucial observation for patients affected with EOEE and/or developmental disabilities.

**Key Points**

- Heterozygous variants in *KCNQ2* or, more rarely, *KCNQ3* genes are responsible for early‐onset developmental/epileptic disorders
- We describe a patient with severe epilepsy and intellectual disability carrying a homozygous frameshift loss‐of‐function variant in KCNQ3
- Familial members who are heterozygous carriers of the *KCNQ3* variant are unaffected
- The present results highlight difference in genetic and pathogenetic mechanisms between *KCNQ2*‐ and *KCNQ3*‐related epilepsies

## 1 INTRODUCTION

Next‐generation sequencing (NGS) technologies have revolutionized diagnostic procedures in developmental disorders, intellectual disability (ID), and pediatric epilepsies, allowing early identification of the molecular defects in an ever‐growing number of human disease‐causing genes.^1^ Early genetic diagnosis is critical for prognostic assessment, genetic counseling, and, in some cases, personalized treatment attempts.^2^

Pathogenic variants in *KCNQ2* and *KCNQ3* genes coding for Kv7.2 and Kv7.3 neuronal voltage‐gated potassium (K^+^) channel subunits cause early‐onset epilepsies with wide phenotypic heterogeneity.^3^, ^4^, ^5^ At the benign end of this spectrum is benign familial neonatal seizures (BFNS), an autosomal dominant self‐limiting neonatal epilepsy with seizures beginning in otherwise healthy infants in the first days of life and spontaneously disappearing in the following months, with mostly normal neurocognitive development and unremarkable neuroimaging.^3^, ^6^, ^7^ On the other hand, sporadic cases of early‐onset epileptic encephalopathy (EOEE) with cognitive disability, various age‐related pathological EEG patterns such as suppression‐burst/multifocal epileptic activity/hypsarrhythmia,^8^, ^9^ and distinct neuroradiological features have been more recently described in association to *KCNQ2* variants.^10^, ^11^

When compared to *KCNQ2*, pathogenic variants in *KCNQ3* have been more rarely described, mostly in families affected with familial forms of benign epilepsies with seizures starting in the neonatal (BFNS)^6^, ^12^, ^13^ or early‐infantile (benign familial infantile seizures, BFIS) period.^14^, ^15^ Fewer than twenty families with BFNS and three families with BFIS with a heterozygous *KCNQ3* pathogenic variant have been reported to date.^16^ In addition, de novo variants in *KCNQ3* have been described in few children with EOEE,^17^, ^18^, ^19^, ^20^ ID apparently without epilepsy,^21^, ^22^ and cortical visual impairment.^23^

Notably, all pathogenic variants in *KCNQ2* and *KCNQ3*, except one,^24^ occur in heterozygosity. In the present manuscript, we report the clinical, molecular, and functional properties of a new *KCNQ3* variant found in homozygous configuration in a 9‐year‐old girl with pharmacodependent neonatal‐onset epilepsy and non‐syndromic ID; the variant, a single‐base duplication in exon 12 of *KCNQ3* (chr8:g.133150233dup in GRCh37; NM_004519.3:c.1599dup), is predicted to result in a frameshift p.(Phe534Ilefs*15) which could lead to either mRNA degradation by the nonsense‐mediated mRNA decay (NMD) machinery^25^ and/or a truncation of a large part of the C‐terminus. Ex vivo results showed that *KCNQ3* transcript levels were markedly reduced in primary fibroblasts from the proband when compared to those from the unaffected noncarrier brother, and in vitro studies revealed that the variant fully abolished the ability of KCNQ3 subunits to assemble into functional homomeric or heteromeric channels with KCNQ2 subunits. The present results provide the first clinical, genetic, and functional evidence for a severe phenotype associated with a homozygous loss‐of‐function (LoF) variant in *KCNQ3* and highlight previously unrecognized difference in genetic and pathogenetic mechanisms between *KCNQ2*‐ and *KCNQ3*‐related epilepsies.

## 2 MATERIALS AND METHODS

### 2.1 Informed patient consent

Written informed consent was obtained from all study participants or from parents/authorized legal guardians. The study was performed within the framework of the Genetique des Anomalies du Développement (GAD) collection performed at Equipe Inserm U1231 of the Université de Bourgogne in Dijon (FR), approved by institutional review board (no DC2011‐1332).

### 2.2 Exome sequencing

Singleton exome sequencing was performed using an Agilent CRE capture kit (Agilent Technologies) and a HiSeq 4000 sequencer (Illumina); exome data analysis and variant filtering were performed as previously described^26^ using the following annotation databases and software versions: dbsnp 149, clinvar 2016_11_03, cosmic COSMICv79, omim 2016_11_29, refseq_annotation 2016_11_27, FASTQC 2015_12_15, TRIMMOMATIC 0.35, BWA_0.7.12, PICARD_2.0.1, GATK_3.5, samtools_1.2, IGVTools_2.3.67, reference genome GRCh37/hg19, refseq 2015_07_30. Reads alignment resulted in coverage of at least 10× for 96.5% of the bases on target and an average sequencing depth of 116.01×.

### 2.3 Cell culture

Chinese hamster ovary (CHO) cells were grown in Dulbecco's Modified Eagle's Medium (DMEM) containing 10% fetal bovine serum (FBS), 2 mmol/L L‐glutamine, penicillin (50 U/mL), and streptomycin (50 μg/mL) in a humidified atmosphere at 37°C with 5% CO_2_. Primary fibroblasts were obtained from punch skin biopsies. Tissue was cut into small pieces (1 × 3 mm), and cells were cultivated in a fibroblast growth medium consisting of AmnioMax (Gibco, Thermo Fisher) supplemented with 20% FBS. For long‐term culture, fibroblasts were maintained in DMEM supplemented with 10% FBS.

### 2.4 Mutagenesis and heterologous expression of KCNQ2 or KCNQ3 cDNAs

The variant of interest was engineered in KCNQ3 human cDNA cloned into pcDNA3.1 (variant 1; NM_004519.3; 872 aa) by QuikChange site‐directed mutagenesis (Agilent Technologies). Channel subunits were expressed in CHO cells by transient transfection using Lipofectamine 2000 (Invitrogen);^27^, ^28^ when two or more cDNAs were cotransfected, their molar ratio was modified such that total cDNA in the transfection mixture was kept constant at 3 μg. Enhanced green fluorescent protein (1 μg; Clontech) was used as transfection marker.

### 2.5 Western blot experiments

KCNQ3 subunits in total protein lysates from CHO cells obtained 24 hours post‐transfection were analyzed by Western blotting on 8% SDS‐PAGE using two primary rabbit anti‐KCNQ3 polyclonal antibodies: (a) the first directed against a C‐terminal epitope (rat aa 668‐686; accession number O88944; C‐KCNQ3) (clone APC‐051, dilution 1:1000; Alomone Labs) and (b) the second raised against an N‐terminal epitope (rat aa 1‐71; N‐KCNQ3) (PA1‐930; dilution 1:1000; Thermo Scientific). Both antibodies also recognized human KCNQ3 subunits. Following exposure to primary antibodies, filters were incubated with HRP‐conjugated anti‐rabbit secondary antibodies (NA934V; dilution 1:5,000; GE Healthcare) and reactive bands visualized by chemiluminescence. Data acquisition and analysis were performed with the Gel Doc Imaging System (Bio‐Rad) and ImageLab software (version 4.1; Bio‐Rad), respectively.

### 2.6 RNA isolation, reverse transcription, and quantitative PCR

Isolation and purification of RNA was performed using the TriReagent (Sigma). 1 μg of total RNA was retrotranscribed with the High Capacity cDNA RT Kit (Applied Biosystem, Thermo Fisher Scientific). For quantitative PCR, cDNA was amplified with the TaqMan Gene Expression assay in a 7500 Fast Real‐Time PCR System thermocycler (Applied Biosystems, Thermo Fisher Scientific). Commercially available probes were used to amplify *KCNQ1, KCNQ2, KCNQ3*, *KCNQ4,* and *KCNQ5* mRNAs (Applied Biosystem TaqMan gene expression, codes *KCNQ1:* hs00923522_m1; *KCNQ2*: hs01548339_m1; *KCNQ3:* hs01120412_m1; *KCNQ4:* hs*00542548*_m1; *KCNQ5:* hs01068536_m1). The comparative ΔΔCT method was used to quantify transcript abundance using the ubiquitin‐conjugating enzyme (UBC; hs05002522_g1) gene as control.^29^ Three separate experiments, each in triplicate, were performed for each probe.

### 2.7 Immunofluorescence

Cells were fixed with 4% paraformaldehyde in PBS for 10 minutes at room temperature (RT). After permeabilization with 0.1% Tween‐20 for 5 minutes and blocking with 0.5% BSA for 1 hour at RT, cells were incubated overnight at 4°C with the N‐KCNQ3 antibody (1:300), followed by a 1 hour incubation with donkey anti‐rabbit Cy3‐conjugated secondary antibody (Applied Biosystems, Thermo Fisher Scientific) at RT. Nuclei were visualized using Hoechst 33258 (1:5000) in PBS. Coverslips were mounted in Fluoromount G (eBioscience, Hatfield, Hertfordshire, UK); images were acquired with a Zeiss inverted LSM 700 confocal laser scanning microscope (Carl Zeiss) and analyzed with ImageJ (NIH). Slides in which the primary antibody was omitted were used as controls in all experiments.

### 2.8 Whole‐cell electrophysiology

Macroscopic current recordings from transiently transfected CHO cells, as well as data processing and analysis, were performed as described.^27^, ^28^ Currents from CHO cells were recorded at room temperature with the whole‐cell configuration of the patch‐clamp technique, using glass micropipettes of 3‐5 MΩ resistance. The extracellular solution contained (in mmol/L) the following: 138 NaCl, 5.4 KCl, 2 CaCl_2_, 1 MgCl_2_, 10 glucose, and 10 HEPES, pH 7.4 with NaOH. The pipette (intracellular) solution contained (in mmol/L) the following: 140 KCl, 2 MgCl_2_, 10 EGTA, 10 HEPES, and 5 Mg‐ATP, pH 7.3‐7.4 with KOH. The pCLAMP software (version 10.0.2) was used for data acquisition and analysis. Linear cell capacitance (C) and series‐resistance (RS) calculation were performed as described previously.^30^ In the experiments with tetraethylammonium, currents were activated by 3‐second voltage ramps from −80 mV to +40 mV at 0.08 Hz frequency. Fast solution exchanges (<1 second) were achieved by means of a cFlow 8 flow controller attached to a cF‐8VS eight‐valve switching apparatus (Cell MicroControls).

### 2.9 Statistics

Data are expressed as the mean ± SEM. Data reported in Table 1 are average values of at least 9 individual measurements, recorded in at least 3 separate experimental sessions (transfections). Statistically significant differences between the data were evaluated with Student's *t* test (*P* < .05).

**Table 1:** Biophysical and pharmacological properties of currents recorded in CHO cells transfected with the indicated plasmid combinations

	cDNA transfected (µg)	n	*V*_½_ (mV)	K (mV/efold)	Current density (pA/pF at 0 mV)	Blockade by 3 mmol/L TEA (%)
Nontransfected	‐	10	‐	‐	0.5 ± 0.1	‐
*KCNQ3*	3	9	−41.9 ± 1.5*, **	8.2 ± 0.9*, **	11.5 ± 4.8*, **	8.0 ± 2.1*, **
*KCNQ3* p.(Phe534Ilefs^*^15)	3	9	‐	‐	0.3 ± 0.1	‐
*KCNQ2 + pcDNA3*	1.5 + 1.5	11	−21.7 ± 1.9	13.2 ± 0.8	21.7 ± 5.1*	‐
*KCNQ2*	3	13	−23.0 ± 1.5	12.0 ± 0.5	42.2 ± 9.7	94.0 ± 1.0**
*KCNQ2* + *KCNQ*3	1.5 + 1.5	23	−35.1 ± 1.6	13.0 ± 0.7	117.6 ± 15.1	56.1 ± 6.6
*KCNQ2* + *KCNQ3* p.(Phe534Ilefs^*^15)	1.5 + 1.5	20	−23.9 ± 1.9**	15.4 ± 1.5	17.5 ± 2.5**	90.0 ± 1.5**
*KCNQ2* +*KCNQ3* + pcDNA3	1.5 + 0.75 + 0.75	13	−27.5 ± 1.0**	13.0 ± 0.7	33.6 ± 6.9**	62.0 ± 4.3*
*KCNQ2* + *KCNQ3* + *KCNQ3* p.(Phe534Ilefs^*^15)	1.5 + 0.75 + 0.75	24	−29.5 ± 1.8**	12.8 ± 0.7	39.6 ± 6.1**	62.2 ± 3.2*

* *P* < 0.05 vs KCNQ2 (3 µg).

** *P* < 0.05 vs KCNQ2 + KCNQ3 (1.5 + 1.5 µg).

## 3 RESULTS

### 3.1 Clinical features

**Figure 1:**
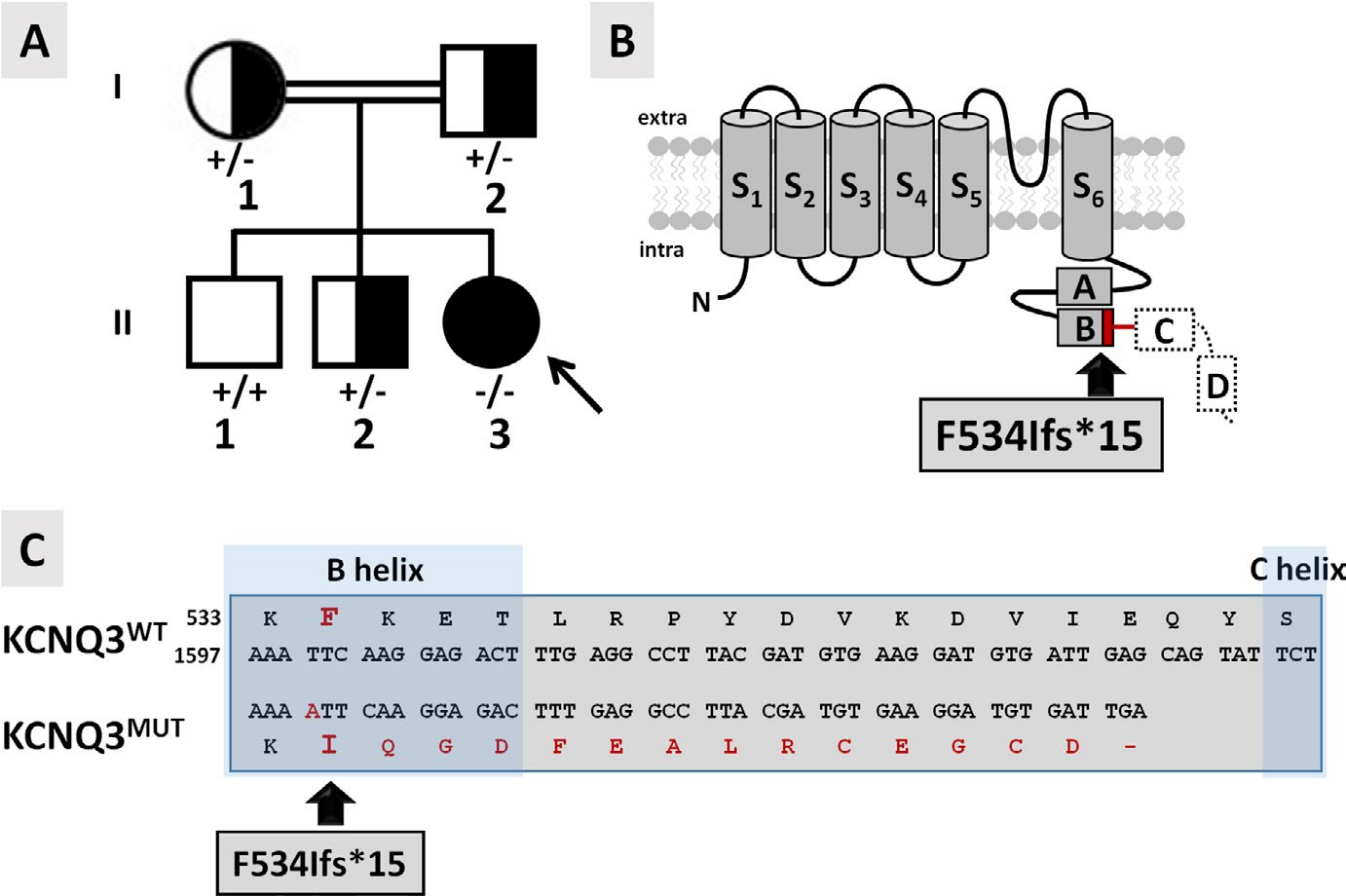
Pedigree of the investigated family and topological models of the mutant KCNQ3 subunit. A, Pedigree of the family investigated. ʻ + ʼ indicates the wild‐type *KCNQ*3 allele; ʻ−ʼ indicates the mutant *KCNQ*3 p.(Phe534Ilefs*15) allele. The arrow indicates the proband. B, Schematic topology of a KCNQ3 subunit: S_1_‐S_6_ refer to the six transmembrane segments, while boxes labeled from A to D depict the four α‐helical regions in the intracellular C‐terminus. The p.(Phe534Ilefs*15) variant located in the helix B is indicated by the arrow. The aa sequence deleted in the mutant KCNQ3 protein is indicated by a dashed line. The red line indicates the amino acid sequence altered by the frameshift variant. C, Partial alignments of the primary sequences of KCNQ3 and KCNQ3 p.(Phe534Ilefs*15, indicated as KCNQ3^MUT^) subunits. The B and C helices are highlighted with darker blue boxes.

The proband (individual II‐3; Figure 1A) is a French 9‐year‐old female originary from Morocco born to consanguineous healthy parents after an uneventful pregnancy and delivery, apart from a transient prematurity risk at 35 weeks. She was delivered at term (38 weeks 4 days), and birth parameters were normal: 3040 g weight, 46.5 cm length, and 34 cm occipito‐frontal head circumference (OCF). At the age of 2 days, she presented with both focal (affecting either the left or right hemi‐body) and generalized convulsions associated with hypotonia, cyanosis, and clonic movements of the four limbs. Biochemical and metabolic screening was noncontributive. Initial neurological examination and tonus were normal. The first electroencephalograms (EEGs), performed in the following days, revealed electrical seizures characterized by central and temporal slow waves prevailing on the right side not always associated with clinical manifestations. Biotin, pyridoxine, and folinic acid were ineffective. At the age of 2 months, convulsions were controlled with phenytoin and vigabatrin; ocular contact was poor; and the tonus was insufficient. At 7 months of age, sodium valproate monotherapy was effective in controlling seizures; a marked strabismus was noted requiring specialized management including botulinum toxin injection. Interruption of sodium valproate treatment at the age of 3‐4 years resulted in seizure recurrence during late night, including febrile episodes, with left hemispheric spikes and waves recorded on the EEG; valproate therapy was therefore reintroduced. Since then, she exhibited rare tonic‐clonic seizures, and all subsequent EEG recordings were normal, the last one at age 6 years. She is currently treated with sodium valproate with good response, and her epilepsy shows the characteristics of a pharmacodependent epilepsy.^31^ Brain MRI performed at day 15 revealed a suspected mild cortical dysplasia of the right frontal and temporal lobes, but a further MRI at age 6 years and 5 months was normal, as well as a brain CT scan. The proband acquired head control at age 6‐7 months, could sit unsupported at age 12 months, and walked independently around age 30‐36 months. At age 3 years, she could speak 2 words and required speech therapy, including Makaton technique while verbal language was insufficient; she attended specialized educational institution at age 5 years and started to produce short rudimentary word associations since age 6 years, with about 10 words in her vocabulary; sentences were still incompletely produced at age 8 years. At age 6.5 years, her psychomotor development was estimated around 22 months. She did not exhibit behavioral disturbances. At last examination (age 9 years), no abnormal morphological features were noted and neurological examination was normal apart from divergent strabismus increased in superior gaze. She has a moderate intellectual disability with poor vocabulary and little autonomy in daily life; she still attends a medical institute with little schooling abilities. Growth charts showed regular evolution of length, weight, and OFC between median and −1 SD. Familial history revealed that a maternal uncle who had mild cognitive disabilities with some degree of learning (reading and writing) difficulties also suffered from transient neonatal seizures, requiring a specialized pediatric follow‐up in the first 6 months of life. However, he has a milder phenotype than his niece as he could achieve a relatively good autonomy in daily life. All other members of the family had normal psychomotor and cognitive development with no history of seizures.

### 3.2 Genetic data

Array‐CGH, *KCNQ2*‐targeted sequencing, and gene panel sequencing in the proband (individual II‐3) were normal; exome sequencing was therefore performed. The analysis on OMIM morbid genes responsible for mental retardation associated or not with epileptic encephalopathy highlighted the presence of two rare heterozygous variants, one in *HERC2*, associated with an autosomal recessive form of mental retardation (MIM 615516), and one in *FRAS1*, associated with autosomal recessive Fraser syndrome 1 (MIM 219000). Both variants were not retained as causative because the mode of inheritance was not compatible with the genotype of our patient. A variant of *DYNC1H1*, gene associated with Mental Retardation autosomal dominant 13 (MIM 614563), was not retained as causal as this variant has been reported 77 times in the healthy population (gnomAD 2.1.1). No further variants were retained using a sporadic mode of inheritance. The homozygosity analysis obtained through HomozygosityMapper^32^ using default parameters revealed relatively small regions of homozygosity in chr1, chr9, chr10, chr14, chr16, and chr22 compatible with the consanguinity between the parents (the 2 grandmothers of the proband are sisters, see expanded pedigree shown as Figure S1). Within these regions, we identified a rare variant of the *DMBT1* gene at the homozygous state, not retained as causal because it was found at the homozygous state in 7 individuals in the healthy population. Outside the regions of homozygosity, the analysis on OMIM morbid genes at the recessive state retained an homozygous variant of the *KCNQ3* gene absent from public databases as the only candidate compatible with the clinical presentation of the proband. This variant (ClinVar submission number SUB5837801) is a homozygous single‐base duplication (chr8:g133150233dup in GRCh37, NM 004519.3:c.1599dup) in exon 12 (135/138 reads detected the variant) which results in a shift in the open reading frame p.(Phe534Ilefs*15) and the occurrence of a premature termination codon (PTC) at position 549, possibly leading to the synthesis of a truncated protein deleted of a large part of the C‐terminal region (Figure 1B,C). The parents and one of the brothers (individuals I‐1, I‐2, and II‐2, respectively) were heterozygous carriers for the *KCNQ3* variant; the eldest brother (individual II‐1) carried two copies of the wild‐type allele (Figure 1A); and the maternal uncle was unavailable for genetic analysis.

### 3.3 The *KCNQ3* mutant allele is expressed at lower levels when compared to healthy control

Premature termination codons often result in mRNA instability and precocious degradation by nonsense‐mediated mRNA decay (NMD).^25^ KCNQs expression was previously reported in human fibroblasts^33^; therefore, the effect of the NM_004519.3:c.1599dup variant on *KCNQ3* transcript levels was evaluated in primary fibroblasts from the proband (II‐3, carrying two copies of the mutant allele) and a control member of the family (II‐1, carrying two copies of the wild‐type allele), using qRT‐PCR. In control cells, the Ct values for the different KCNQs are the following: KCNQ1: 36; KCNQ2: 28; KCNQ3: 28; KCNQ4: 32; and KCNQ5: 31; all these values are considerably higher than that of UBC, the housekeeping gene used as comparator (Ct value of 25), consistent with the described low expression levels for these transcripts.^33^

**Figure 2:**
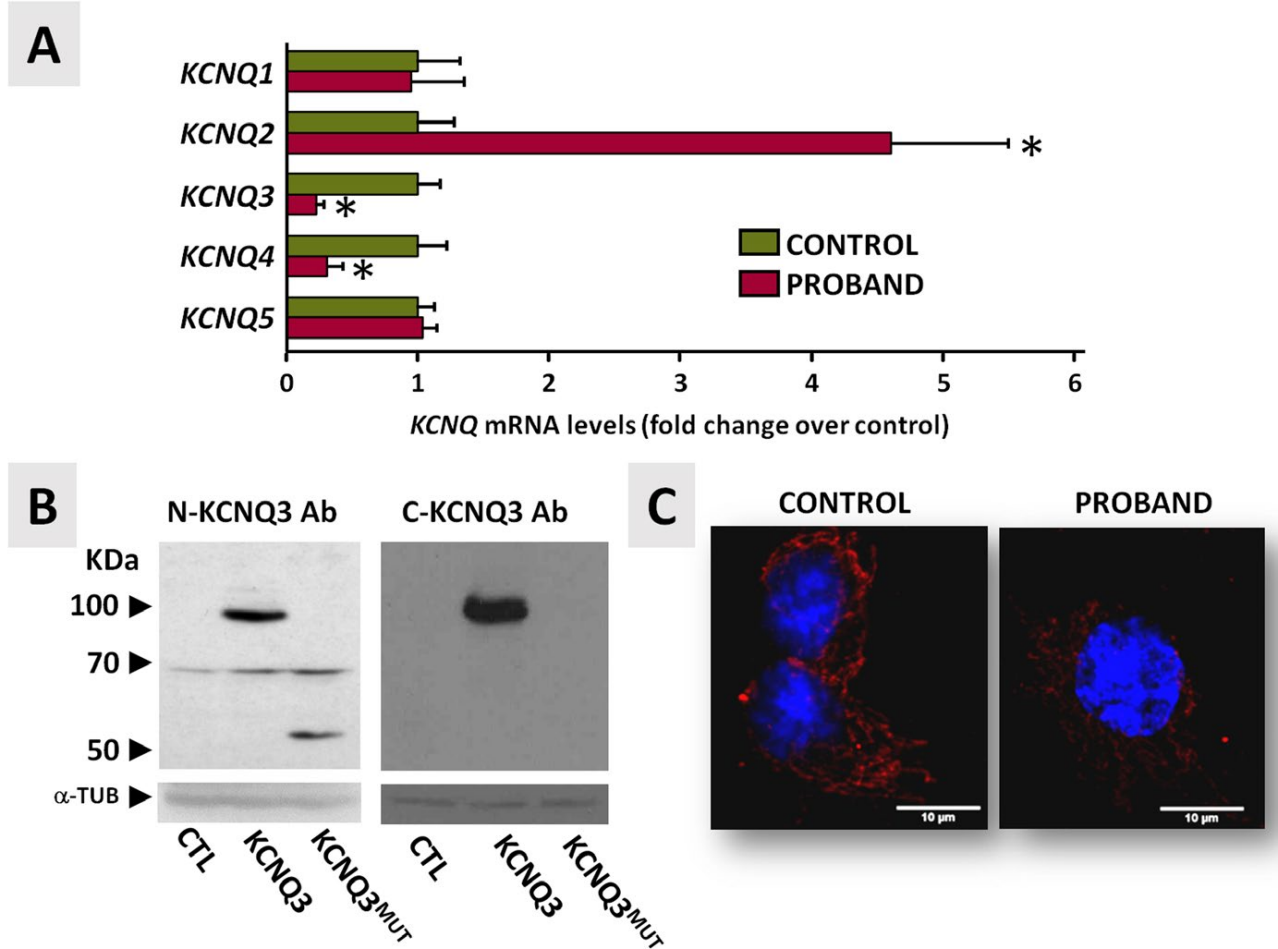
KCNQ transcript and protein expression profile in primary fibroblasts from the proband (individual II‐3) and healthy brother (individual II‐1). A, *KCNQ*1‐5 qRT‐PCR data from primary fibroblasts. Data are expressed as cycle threshold values for each *KCNQ* transcript normalized to that of *UBC;* after normalization, data from control fibroblasts were expressed as one (green bars), and data from proband fibroblasts were expressed relative to controls (red bars). Asterisks indicate values statistically different (*P* < 0.05) from respective controls. B, Western blot performed on protein lysates from transiently transfected CHO cells using N‐ and C‐KCNQ3 antibodies. CHO cells were transfected with wild‐type (KCNQ3) or mutant (KCNQ3^MUT^) *KCNQ3* cDNA, and total lysates were analyzed with N‐KCNQ3 (left panel) or C‐KCNQ3 (right panel) antibodies. α‐tubulin (α‐TUB) served as a loading control. C, Confocal images of fibroblasts from the proband (individual II‐1) and the healthy brother (individual II‐1) stained with N‐KCNQ3 primary antibodies (in red) and a nuclear marker (Hoechst, in blue)

As shown in Figure 2A, in cells from the proband, KCNQ3 transcript levels were reduced to ~22% when compared to those in control cells; a quantitatively similar decrease in transcript levels was also observed for KCNQ4, whereas those for KCNQ2 were markedly increased. No significant difference was instead observed when comparing KCNQ1 and KCNQ5 transcript abundance between proband and control primary fibroblasts.

To investigate whether such decrease in KCNQ3 mRNA levels also led to changes in KCNQ3 protein expression, immunofluorescence experiments using N‐KCNQ3 primary antibodies were performed in primary fibroblasts from the proband and the noncarrier, unaffected brother. These antibodies were validated in Western blots on lysates from CHO cells transiently transfected with wild‐type KCNQ3 or KCNQ3 p.(Phe534Ilefs*15)‐encoding plasmids (KCNQ3^MUT^; Figure 2B, left panel). In these experimental groups, N‐KCNQ3 antibodies specifically recognized a ~100 kDa or a ~60 kDa protein band, respectively. These values are consistent with the molecular masses expected for wild‐type or mutant KCNQ3 proteins, respectively. Western blot experiments also revealed that, in this heterologous cellular system, the mutant protein was expressed at lower levels when compared to the wild‐type KCNQ3 protein (Figure 2B, left panel). As expected, C‐KCNQ3 antibodies targeting a C‐terminal epitope located downstream the predicted premature termination site introduced by the variant, while detecting the 100kDa band corresponding to the wild‐type KCNQ3 protein, failed to recognize any specific signal in cells transfected with the mutant plasmid (Figure 2B, right panel). Western blot experiments performed in primary fibroblasts from proband and control samples with both antibodies failed to detect specific signals corresponding to either wild‐type or mutant KCNQ3 proteins, possibly because of the low abundance of the endogenous protein in these cells (data not shown). Immunochemistry experiments using N‐KCNQ3 antibodies performed in primary fibroblasts from the proband (individual II‐3) and the wild‐type brother (individual II‐1) (Figure 2C, right and left panels, respectively) revealed a KCNQ3‐specific signal in both groups, although the intensity was lower in the former when compared to the latter. Notably, in fibroblasts from both individuals, the immunofluorescent signal was mainly cytosolic, showing a subcellular distribution consistent with an endoplasmic reticulum localization.

### 3.4 Functional and pharmacological characterization of homomeric and heteromeric channels carrying KCNQ3 p.(Phe534Ilefs*15) mutant subunits

Previously shown data obtained in primary fibroblasts from the proband suggest that the p.(Phe534Ilefs*15) truncating variant led to a significant decrease in KCNQ3 transcript and protein levels, a result consistent with NMD.^25^ However, transcript expression pattern in fibroblasts might not parallel that in neurons; moreover, a significant, although reduced in abundance, fraction of KCNQ3 protein was still detected in fibroblasts. Therefore, electrophysiological recordings in transiently transfected CHO cells were carried out to evaluate the effects prompted by the p.(Phe534Ilefs*15) variant on KCNQ3 channel function.

**Figure 3:**
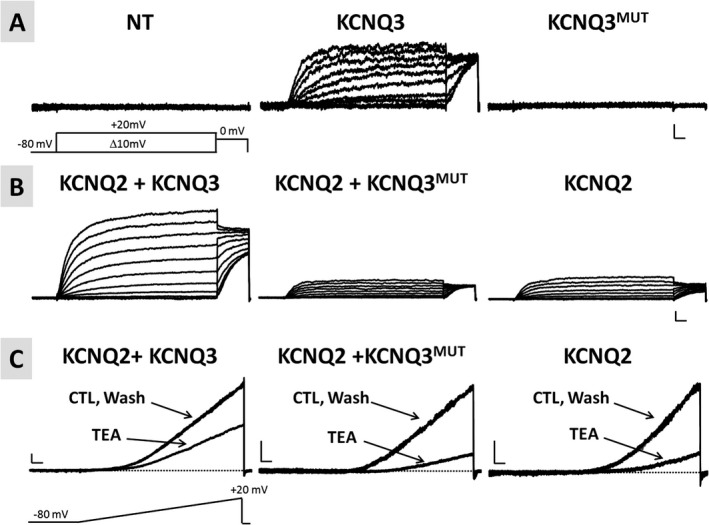
Functional characterization of homomeric or heteromeric channels incorporating KCNQ3 p.(Phe534Ilefs*15) subunits. A, Representative current traces from nontransfected cells (NT; left panel) or from cells transfected with either wild‐type KCNQ3‐ (KCNQ3; middle panel) or KCNQ3 p.(Phe534Ilefs*15)‐encoding plasmids (KCNQ3^MUT^; right panel) in response to the voltage protocol shown. Horizontal scale bar: 100 ms; vertical scale bar: 2 pA/pF. B, Representative current traces recorded in cells expressing the indicated subunits, in response to the same voltage protocol shown in A. Horizontal scale bar: 100 ms; vertical scale bar: 20 pA/pF. C, Representative current traces from cells expressing the indicated subunits in response to the indicated voltage ramp protocol before TEA exposure (CTL, control), during TEA exposure (TEA, 3 mmol/L), and upon drug washout (Wash). Horizontal scale bar: 200 ms; vertical scale bar: 10 pA/pF

Heterologous expression of wild‐type KCNQ3 subunits led to the appearance of voltage‐dependent K^+^‐selective currents characterized by a slow time course of activation and deactivation and a threshold for current activation around −50 mV.^5^, ^7^ By contrast, no currents could be recorded in cells transfected with the KCNQ3 p.(Phe534Ilefs*15) plasmid, consistent with the variant causing a complete loss‐of‐function (LoF) effect (Figure 3A and Table 1).

In adult neuronal cells, KCNQ3 subunits assemble into heteromeric channels with KCNQ2 to form I_KM_.^5^ To investigate the functional consequences of mutant KCNQ3 subunits when coexpressed with KCNQ2 subunits, CHO cells were cotransfected with KCNQ2 and KCNQ3 cDNAs at a 1:1 ratio (to mimic the genetic balance of the noncarrier Individual II‐1) or with KCNQ2 and KCNQ3 p.(Phe534Ilefs*15) at 1:1 ratio (to mimic the genetic balance of the proband, Individual II‐3). Coexpression of wild‐type KCNQ3 subunits and KCNQ2 subunits markedly increased current size when compared to KCNQ2‐ or KCNQ3‐only expressing cells (Table 1); in addition, KCNQ2/3 heteromeric channels display a significant leftward shift in the activation *V*_1/2_ and a decrease in current sensitivity to blockade by TEA (Table 1).^5^, ^28^, ^34^ By contrast, coexpression of KCNQ3 p.(Phe534Ilefs*15) with KCNQ2 subunits failed to enhance current density and to modify the *V*_1/2_ (Figure 3B and Table 1). Currents recorded in KCNQ2 + KCNQ3 p.(Phe534Ilefs*15)‐transfected cells displayed a sensitivity to blockade by 3 mmol/L TEA higher than that of KCNQ2/KCNQ3‐transfected cells and identical to that of KCNQ2‐only transfected cells (Figure 3C, Table 1). These results suggest that mutant KCNQ3 subunits fail to form functional heteromeric channels with KCNQ2 subunits.

To replicate in vitro genetic combination occurring in the family members who are heterozygous carriers of the mutant allele (Individuals I‐1, I‐2, and II‐2), functional studies were also carried out upon transfection of KCNQ2, KCNQ3, and KCNQ3 p.(Phe534Ilefs*15) plasmids in a 1:0.5:0.5 cDNA ratio; for these experiments, cells transfected with an identical (1:0.5:0.5) cDNA ratio of KCNQ2 + KCNQ3+pcDNA plasmids served as controls. The results obtained suggest that the presence of an halved dose of functional/wild‐type KCNQ3 allele is sufficient to generate currents displaying the biophysical and pharmacological properties of KCNQ2/KCNQ3 heteromers, although with a reduced density when compared to that recorded in cells transfected with a full dose of KCNQ3 (1:1 KCNQ2:KCNQ3 cDNA ratio). Notably, the pharmacological and functional properties of the currents recorded in cell transfected with KCNQ2, KCNQ3, and KCNQ3 p.(Phe534Ilefs*15) plasmids (1:0.5:0.5 cDNA ratio) were identical to KCNQ2 + KCNQ3+pcDNA‐expressing cells (1:0.5:0.5 cDNA ratio) (Table 1).

## 4 DISCUSSION

### 4.1 Epilepsy and ID caused by a novel homozygous KCNQ3 frameshift variant: clinical and ex vivo results

We herein report the clinical, ex vivo, and in vitro results from a family carrying the novel frameshift p.(Phe534Ilefs*15) variant in *KCNQ3* (NM_004519.3:c.1599dup). The proband is a 9‐year‐old girl diagnosed with neonatal‐onset and pharmacosensitive seizures and non‐syndromic ID; she was found to be a homozygous carrier for this variant. While our study was in progress, another family with three siblings affected with neonatal‐onset seizures (reported as pharmacosensitive in one of them) and ID of variable severity due to a different homozygous frameshift variant in *KCNQ3* (c.*1220_1221delCT*; p.(Ser407Phefs*27)) has been described in a large cohort of children suffering from epileptic encephalopathy.^24^ In this family, where no functional analyses were performed, neonatal seizures or neurodevelopmental problems did not occur in consanguineous parents or extended family members who were heterozygous carriers for the *KCNQ3* mutant allele. One of the affected sibling in Kothur et al.^24^ exhibited severe developmental delay, while the two others presented with mild ID; since genetic analysis was only limited to a restricted epileptic encephalopathy gene panel, it is unknown whether additional genetic defects, potentially enabled by consanguinity, could account for the more severe phenotype in this particular sibling.

PTC‐containing mRNAs often undergo NMD when the PTC is located upstream the ~50‐55th nucleotide before the last exon‐intron junction,^35^ as it occurs with the presently described variant. NMD represents a quality control mechanism to avoid production of truncated proteins with potentially deleterious effects.^25^, ^36^ *KCNQ3* transcript levels in primary fibroblasts^33^ from the proband were markedly decreased when compared to those from the unaffected noncarrier brother II‐1, a result consistent with NMD; the reduction in KCNQ3 mutant transcript levels was accompanied by an increase in KCNQ2, a reduction of KCNQ4, and no change in KCNQ1 or KCNQ5 transcript levels. It remains to be determined whether these changes are due to compensatory effects or uncover a more complex coordination of gene expression. In this regard, whether the transcriptional repressor RE1 silencing transcription factor (REST), which has been shown to regulate KCNQ2 and KCNQ3^37^ as well as KCNQ4^38^ expression, participates in this coordination is currently unknown; moreover, it should be highlighted that conditional and selective ablation of Kcnq2 or Kcnq3 in cortical mouse tissue also modifies the expression of other members of the Kcnq subfamily.^39^ Notably, immunofluorescence experiments also revealed a reduced intensity of the KCNQ3 signal in primary fibroblasts from the proband; in these experiments, the KCNQ3 signal showed a diffuse cytoplasmic staining consistent with endoplasmic reticulum‐Golgi localization with no remarkable difference in subcellular localization between fibroblasts from the proband or the control brother. A diffuse staining pattern on both cell surface and intracellular components was also detected for KCNQ3 in rat^40^ and human^41^ hippocampal and cortical pyramidal neurons.

### 4.2 Functional consequences of the KCNQ3 p.(Phe534Ilefs*15) variant

Our results indicate that transcript and protein levels encoded by the mutant *KCNQ3* allele are detectable, prompting investigation of the functional consequences of the p.(Phe534Ilefs*15) variant on KCNQ3 subunit function. The results obtained clearly suggest the KCNQ3 p.(Phe534Ilefs*15) protein is fully nonfunctional; indeed, homomeric expression of mutant subunits failed to generate detectable voltage‐gated K^+^ currents. Moreover, mutant KCNQ3 subunits did not incorporate into functional heteromeric channels when coexpressed with KCNQ2 subunits, a result consistent with the variant‐induced deletion of a significant portion of the long C‐terminus where critical domains responsible for homomeric and heteromeric subunit assembly and plasmembrane trafficking have been identified.^4^, ^42^, ^43^ Furthermore, the presence of an halved dose of wild‐type KCNQ3 protein, an experimental condition mimicking in vitro the genetic combination of the family members who are heterozygous carriers of the mutant *KCNQ3* allele, was sufficient to generate currents displaying the biophysical and pharmacological properties of KCNQ2/Q3 heteromers, although with a reduced density when compared to that recorded in cells transfected with a full dose of KCNQ3. Currents recorded in cells transfected with KCNQ2, KCNQ3, and mutant KCNQ3 cDNAs were identical to those in cells transfected with the same cDNA amount of KCNQ2 and KCNQ3 cDNAs only, arguing in favor of the inability of the protein encoded by the mutant allele to heteromerize and interfere with the function of wild‐type subunits. This functional result strongly suggests that haploinsufficiency is the main molecular mechanism for the severe disease in the affected proband; this is in sharp contrast to *KCNQ2*‐related EOEE pathogenesis, where mutant subunits carrying heterozygous missense variants are functional and heteromerize with wild‐type subunits, thereby poisoning channel function by a dominant‐negative mechanism.^27^, ^44^ Additional pathogenic mechanisms for KCNQ2 EOEE include changes in subcellular localization,^45^ and/or in calmodulin‐46, 47 or phosphatidylinositol 4,5‐bisphosphate^48^‐dependent regulation.

### 4.3 Clinical spectrum and genetic mechanisms of KCNQ3‐related diseases

Heterozygous pathogenic variants in *KCNQ3* have been associated with neonatal‐onset epilepsies showing broad clinical heterogeneity and diverse genetic transmission mechanisms.^16^ These range from relatively benign familial phenotypes with seizures starting in the neonatal (BFNS)^6^, ^12^, ^13^ or early‐infantile (BFIS)^14^, ^15^ period, to sporadic cases with severe clinical presentations characterized by developmental disabilities with or without refractory seizures^17^, ^19^, ^21^, ^22^, ^49^ or by cortical visual impairment.^23^

Notably, in both familial and sporadic cases, *KCNQ3* pathogenic variants are all single missense variants, either with autosomal dominant inheritance or arising de novo, respectively (34/34; www.rikee.org).^11^ A notable exception is a recently described EOEE patient who carries two missense variants in compound heterozygosity, each inherited from an asymptomatic parent.^20^ Interestingly, no heterozygous pathogenic frameshift *KCNQ3* variant has ever been associated with a human phenotype, whereas haploinsufficiency due to heterozygous frameshift variants in *KCNQ2* is a frequent cause of BFNS;^16^, ^50^ indeed, frameshift/deletion variants account for 36% (39/108) of BFNS‐causing variants in the KCNQ2 gene^11^, ^13^, ^51^, ^52^ (www.rikee.org). Notably, in both families where *KCNQ3* homozygous frameshift variants were identified, that is, the presently described family and the one reported by Kothur et al.^24^, heterozygous carriers of the *KCNQ3* frameshift variant are unaffected, with no seizures or psychomotor/cognitive impairment. Instead, no homozygous frameshift variant in *KCNQ2* has ever been described in humans, as minimal KCNQ2 residual activity is probably essential under penalty of potential lethality. Although several potential mechanisms may account for the more severe clinical consequences associated with *KCNQ2* variants when compared to *KCNQ3* ones, the fact that, in both rodents and human brains,^53^, ^54^ the ratio of KCNQ3 to KCNQ2 expression is low at birth and increases during postnatal development provides a plausible explanation; notably, deletion of Kcnq2, but not of Kcnq3, from cortical pyramidal neurons in mice is sufficient for the development of aberrant EEG activity and leads to death by the third week of life.^39^

Noteworthy, about 10 nonsense or frameshift variants leading to KCNQ3 truncation spread throughout the gene are reported in public databases such as ClinVar^55^or gnomAD.^56^Among these variants, only one, found in an individual in the gnomAD’s non‐neuro‐samples from individuals who were not ascertained for having a neurological condition in a neurological case/control study, was interpreted as pathogenic (although the phenotype is unknown), while the others were associated with an uncertain clinical significance.

## 5 CONCLUSIONS

This is the first study exploring the functional consequences of a novel *KCNQ3* homozygous LoF variant responsible for a severe phenotype characterized by neonatal‐onset pharmacodependent seizures, with developmental delay and ID. Ex vivo and in vitro experiments revealed a decrease in transcript abundance proportional to variant expression levels and an impaired ability of mutant subunits to assemble into functional homomeric or heteromeric channels with KCNQ2. A lesser degree of channel dysfunction occurs when a single copy of the mutant allele is present, a result possibly contributing to the lack of neurodevelopmental phenotype in heterozygous carriers. The described LoF mechanism allows to hypothesize that, in close analogy to LoF variants found in *KCNQ2*‐EOEE‐affected patients,^50^, ^57^ KCNQ activators such as retigabine may be useful precision medicines to counteract the channel dysfunction triggered by the herein described novel *KCNQ3* variant as well as similar variants that may be identified in the future.

## CONFLICT OF INTEREST

The authors declare no competing financial interests. The Authors confirm that they have read the Journal's position on issues involved in ethical publication and affirm that this report is consistent with those guidelines.
