# Supplementary material for: A novel homozygous KCNQ3 loss‐of‐function variant causes non‐syndromic intellectual disability and neonatal‐onset pharmacodependent epilepsy
Source: Epilepsia Open. 2019 Aug 11;4(3):464–75. doi: 10.1002/epi4.12353 (PMC6698674; doi:10.1002/epi4.12353)
Supplement: Supplementary file 1 [file EPI4-4-464-s001.pptx]

## Slide 1
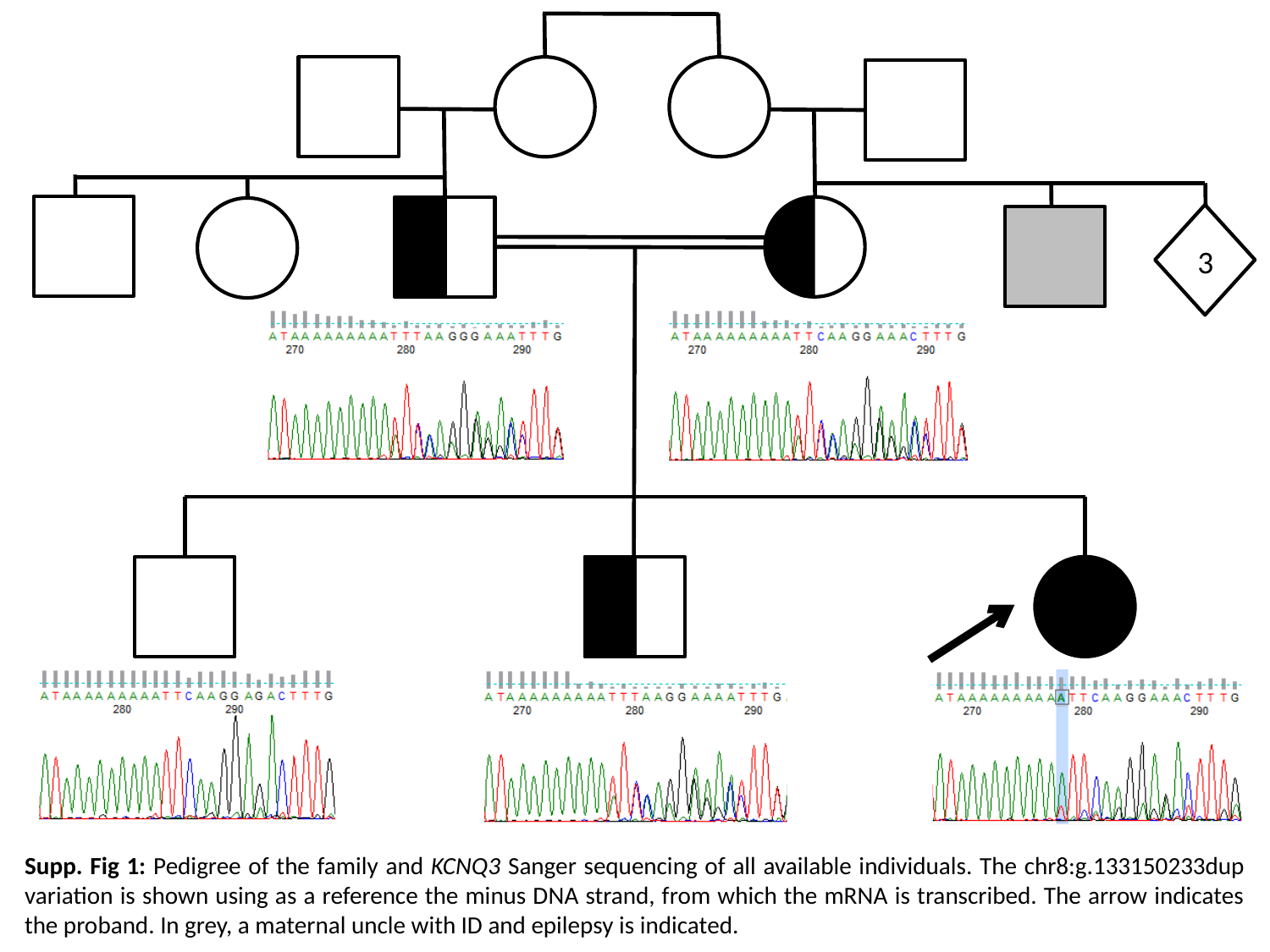

3
Supp. Fig 1: Pedigree of the family and KCNQ3 Sanger sequencing of all available individuals. The chr8:g.133150233dup variation is shown using as a reference the minus DNA strand, from which the mRNA is transcribed. The arrow indicates the proband. In grey, a maternal uncle with ID and epilepsy is indicated.
